# The Greek version of Shoulder Pain and Disability Index (SPADI): translation, cultural adaptation, and validation in patients with rotator cuff tear

**DOI:** 10.1007/s10195-016-0425-8

**Published:** 2016-09-13

**Authors:** S. Vrouva, C. Batistaki, E. Koutsioumpa, D. Kostopoulos, E. Stamoulis, G. Kostopanagiotou

**Affiliations:** 1 2nd Department of Anesthesiology, School of Medicine, National and Kapodistrian University of Athens, “Attikon” University Hospital, 1 Rimini Str, Athens, 12462 Greece; 2 Department of Physical Therapy, 401 Army General Hospital of Athens, Athens, Greece; 3 Intensive Care Unit, University Hospital of Larissa, Larissa, Greece; 4 1st Department of Orthopedics, 401 Army General Hospital of Athens, Athens, Greece; 5 Department of Radiology, 401 Army General Hospital of Athens, Athens, Greece

**Keywords:** Shoulder pain, Disability, SPADI

## Abstract

**Background:**

This study aimed to translate and culturally adapt a Greek version of the Shoulder Pain and Disability Index (SPADI) questionnaire and to validate its usage in Greek patients.

**Materials and methods:**

A forward and backward translation was performed, and the final version of the Greek questionnaire was administered to 134 outpatients (mean age 47.4 ± 14.5) with rotator cuff tear under conservative treatment. The questionnaire was re-administered 2–5 days later to assess test–retest reliability. Patients completed the Greek SPADI, the Greek version of the Quick DASH (Disability of the Arm, Shoulder and Hand Questionnaire) and the EuroQoL EQ-5D. 102 of the 134 questionnaires were considered valid.

**Results:**

The internal consistencies of the SPADI total and its subscales measured with Cronbach’s alpha coefficient were high (0.932 for SPADI-Total, 0.899 for SPADI-Disability, 0.905 for SPADI-Pain). Intraclass correlation coefficients showed excellent test–retest reliability (0.899 for Disability, 0.902 for Pain, and 0.929 for total SPADI). A significantly high positive correlation was found between the SPADI total score and its subscales, and Quick DASH for Pain and Disability. Significant correlations were also found between SPADI scales and EQ-5D variables. There was a moderate positive correlation with the variables “self-reliance” (*r* = 0.66), “common activities” (*r* = 0.58), and “pain/discomfort” (*r* = 0.49), and a weaker correlation with the “mobility” variable (*r* = 0.20). Factor analysis (PAF method) revealed a bidimensional formation of the SPADI. Eight items (five pain/three disability) weighted the first factor by >0.5, and five disability items weighted the second factor.

**Conclusions:**

The Greek SPADI represents a valid and reliable tool for measuring pain and disability in patients with painful shoulder disorders.

**Level of evidence:**

Level 3.

## Introduction

Shoulder pain has a significant cost for health care and a serious impact on quality of life which influences the social and the working aspects of living [1]. An estimated 19 % of the adult population in Europe seems to experience moderate to severe pain in the shoulder joint area [1], which has consequences on daily living [2]. The main cause of shoulder pain is related to rotator cuff problems [3, 4], with an incidence of 20.7 % in the general population which increases with age [5, 6–9]. Hermans et al. [7] in a recent meta-analysis reported that the incidence of rotator cuff tear ranges from 33 to 81 %. In patients with partial rotator cuff rupture, there is usually a limitation of the range of motion, which includes mainly rotational movements (medial–lateral) and abduction [10]. Pain is common during the night, in addition to muscle weakness during shoulder elevation [11]. Itoi et al. [1] report that the largest percentage of patients who decide to seek medical help complain mostly about pain, while a smaller percentage experience both pain and muscle weakness.

One of the easiest ways to obtain information about musculoskeletal pain is through the use of appropriately designed, self-assessment questionnaires, which collect specific information from the participants and are also used as patient-reported outcome measures (PRO) [1]. Μore than 30 different questionnaires about pain in the shoulder area have been described in the literature [13]. The most commonly used are the DASH (Disability of the Arm, Shoulder and Hand) Questionnaire, the Shoulder Pain and Disability Index (SPADI), the American Shoulder and Elbow Surgeons (ASES) Society standardized shoulder assessment form, the Shoulder Disability Questionnaire (SDQ), and the Western Ontario Shoulder Instability Index (WOSI) [13], as well as the Constant (Murley) Score (CS), the Simple Shoulder Test (SST) and the Oxford Shoulder Test (OST) [14]. The SPADI is considered to be, by comparison, one of the most useful instruments about the shoulder joint, and has been tested in various clinical settings [13, 15, 18–22]. It is self-completed, and assesses both shoulder pain and dysfunction [8–22]. Translation and cultural adaptation of the SPADI questionnaire has been performed into many other languages, such as German [23], Portuguese [24], Arabic [25], Tamil (Indian) [26], Turkish [27], and Slovene [28], in order to detect pain and functional status of patients with non-specific shoulder pain. Turkish researchers described the correlation of SPADI with records of the range of motion of the joint, as well as quality of life measured with the SF36 [27]. The same applies to German researchers, who studied the applicability of SPADI in patients undergoing shoulder arthroplasty [23]. In addition, much literature exists regarding multiple testing of factor analysis of the SPADI questionnaire, aiming to determine its validity in various shoulder pain states [29–31]. However, until today, no Greek version of the SPADI questionnaire exists.

Therefore, the aim of this study was to translate and culturally adapt a Greek version of the SPADI questionnaire and to validate its usage in Greek patients with partial rotator cuff tear. For that reason, we performed a thorough investigation of the SPADI scale, in terms of internal consistency (reliability) and validity (both construct and structure validity), in a population-based study of patients with self-reported symptoms related to their shoulders.

## Materials and methods

### Shoulder Pain and Disability Index (SPADI)

The SPADI is a self-administered questionnaire [30] created to assess shoulder pain and dysfunction [20]. It consists of 13 items that assess two different areas [20]. The first five items measure the pain, and the next eight items assess patients’ disability [10]. The SPADI questionnaire has been issued in two different forms [22]: the first version requires completion of a visual analogue scale (VAS) [31], while the second version has a ten-point numerical rating scale (NRS) [22, 32]. In the latest version, which was used in this study, the questionnaire was developed in order to facilitate its use by the patient [22]. The patient answers the questions depending on the degree that corresponds to their pain and difficulty in movement, on a numerical rating scale ranging from 0 (for no pain and difficulty) to 10 (for maximum pain, and such difficulty so that the patient needs help) [20, 22]. The final score is derived by summing the individual responses and reducing them into a percentage (%). The time required to complete the questionnaire ranges between 5 and 10 min [22].

### EQ-5D

The EuroQoL (EQ)-5D is a widely used questionnaire developed in order to record information about quality of life of a certain population [33]. It has been translated into and used in many languages, including Greek [33, 34], in order to collect information related to the state of health in the general population, or in groups suffering from a particular disease [33, 35]. It consists of two main parts [36, 42]: the first is a five-dimensional descriptive system, with questions related to mobility, self-care, usual activities, pain/discomfort, and anxiety/depression [36, 37], where the patient has to select one out of five answers for each question ranging from “I have no problems…” to “I am unable to…” [37, 42]. The response to each of the five dimensions is numerical, ranging from 1 to 5, with “5” representing the most severe problem [37, 42]. A visual analogue scale (0–100) comprises the second part of the questionnaire, where the patient self-reports their current health status from “0” (representing the worst possible health) to “100” (representing excellent health) [36, 37, 42]. The EQ-5D measure requires about 2 min to complete [37].

### Quick Disabilities of the Arm, Shoulder and Hand questionnaire (quick DASH)

The DASH was advanced by the Institute for Work and Health and the American Academy of Orthopaedic Surgeons (AAOS) [38]. It was created in order to discriminate and evaluate the physical disability and the symptoms of patients with musculoskeletal disorders of the upper extremity [39]. The ability to perform an activity is measured, regardless of how it is executed by the patient [39]. The primary part of the questionnaire, the Quick DASH disability/symptoms score, consists of 11 components [38], and each component is scored on a five-point ordinal scale [39]. All of the resulting responses are summed and averaged in order to calculate the total DASH score [38]. This value is reduced by one and then multiplied by 25, and provides a total score that ranges from best to worst on a scale of 0–100 [38] (with 100 being the worst score) [38]. The calculation demands completion of at least 10 of the 11 components [38].

### Translation and cultural adaptation of the SPADI questionnaire

The study took place after obtaining approval by the Ethics Committee of both the 401 Army General Hospital of Athens and the “Attikon” University Hospital. The linguistic validation process was initiated after communication with the original developer of the questionnaire [20], in order to acquire consent. In the first phase, translation of the questionnaire from English to Greek was made according to international guidelines [40]. The objective of the translation was not a word-for-word match, but the Greek conceptual performance of the queries. The process stipulates that three independent translators with Greek as their native language and an advanced level in English language translated the questionnaire into Greek (“translation forward”). The translators were a physiotherapist, an orthopaedic surgeon and a professor of English language and literature. Subsequently, they took into consideration the three translations, and assembled a final form of the Greek questionnaire [41]. Afterwards, a fourth researcher, who had English as his native language, translated the Greek questionnaire into English (“translation backwards”) [41]. Finally, when comparing the two questionnaires, discrepancies had not arisen, and the final form of the Greek questionnaire was distributed to 30 patients for pilot testing [41]. Since the participants stated in interviews that they had no trouble understanding and answering the questions, alterations were not made and the Greek SPADI version was then validated.

### Participants

Informed consent was obtained from all individual participants included in the study. The study has been approved by the ethics committee of the institutions involved, and the ethical standards are in accordance with the Declaration of Helsinki. The participants were all Greek citizens, patients of the 401 Army General Hospital of Athens and the “Attikon” University Hospital, aged 20–80 years, and suffering from a rotator cuff tear of more than 3 months duration. The rotator cuff tear was confirmed by clinical testing combined with magnetic resonance imaging (MRI) and ultrasound. All patients had been treated conservatively. Patients had a negative history of neurological and psychiatric issues, and they had not undergone previous surgery on the affected or the ipsilateral shoulder. The questionnaire was administered to 134 patients, but only 102 questionnaires were considered valid, with all questions answered. These patients re-completed the questionnaire within 2–5 days.

### Medical history and demographic characteristics

The first section of the study collected information related to sex, age, weight, presence of coexisting diseases, pharmacological treatment of these diseases, and recording of the affected and the dominant upper limb. The patients also completed two numerical analogue scales. One was about the previous week’s pain and the other evaluated the pain while filling the questionnaires. The second part of the study comprised the completion of the Greek version of the EQ-5D [42] in order to evaluate the quality of patients’ lives.

### Statistical analysis

The statistical analysis was performed using SPSS 17.0 for Windows (SPSS Inc, Chicago, IL, USA) [9]. *P* values less than 0.05 were considered statistically significant [39]. The SPADI scores were tested by the Kolmogorov–Smirnov test of normality, and a *p* value of 0.2 was obtained (>0.05), showing acceptance of the null hypothesis (that SPADI scores were normally distributed).

To examine whether the difference between men and women in the total SPADI scores was statistically significant, the *t* test was performed for the equality of means between men and woman and the hypothesis was rejected evidently (*p* > 0.05).

In order to evaluate differences in SPADI scores regarding different functional status and different ages of patients, we classified the total SPADI score into four classes (0–25, 25–50, 50–75, and 75–100), and age into three different subgroups (20–40, 40–60, and >60 years old).

### Reliability

The internal consistency of the SPADI scale and the EQ-5D questionnaire was assessed using Cronbach’s alpha coefficient, which represents a measure of how well each question (item) of the scale is correlated with the sum of the remainders. Values of Cronbach’s alpha equal to or greater than 0.7 indicate good reliability, while values >0.9 indicate excellent reliability [23, 24, 43].

In order to quantify the test–retest reliability or the stability over time, the intraclass correlation coefficient (ICC) was used (i.e. the degree to which the same test results are acquired for repeated assessments, although no actual change is predicted in the intervening period) [23]. The ICC was determined for the agreement between the two (test and retest) responses for the SPADI subscales (pain and disability), for the total SPADI score, and also for comparison of these values with those of other researchers [23]. The ICC can range from 0 (no agreement) to 1 (perfect agreement) [23], and according to Fleiss’ [39] classifications ICCs >0.75 signify exemplary reliability, values ranging between 0.4 and 0.75 acceptable to good reliability, and values <0.4 indicate poor reliability [11, 39].

### Validity

The construct validity of the SPADI score was examined by determining how well SPADI scores correlated with those other instruments, such as the Quick DASH [23, 38]. As suggested by Rowntree [39], correlation coefficients below 0.2 were considered very feeble or imperceptible; between 0.2 and 0.4 feeble or low [39]; between 0.4 and 0.7 average [39]; between 0.7 and 0.9 firm or high [39]; and above 0.9 very strong or very high [11]. Undoubtedly, high correlations are expected among instruments with similar designs (e.g. the SPADI and the DASH), verifying construct validity. All correlations were determined using Pearson’s correlation coefficient.

Structural validity refers to the degree to which a measure evaluates the domain of concern of the SPADI and was inspected through factor analysis [30] (a statistical technique used on a group of items in order to determine whether the items from coherent subsets are self-sufficient from one another). In order to discover underlying factors or dimensions of the SPADI scale, our data (102 patients) passed the Bartlett’s Test of Sphericity (*p* value <0.001), and so items were analyzed by factor analysis (FA) with the extraction method of principal axis factoring (PAF) with Varimax Rotation. Factors were elicited according to the Kaiser criterion of maintaining eigenvalues larger than 1 [30]. In PAF, the analysis of data structure focuses on shared variance and not on sources of error that are unique to individual measurements.

## Results

### Descriptive statistics

One hundred and thirty-four patients were studied, resulting in 102 valid questionnaires. The sample consisted of 41.2 % (*n* = 42) men and 58.8 % (*n* = 60) women, of mean age 47.4 ± 14.5 years (range 20–80 years). Descriptive characteristics of patients revealed that 52 % of them had a higher level of education, 13.7 % post-secondary education, and 22.5 % and 4.9 % higher and lower secondary education, respectively, and only 6.9 % had a primary level of education. The mean values of SPADI scores and Quick DASH for the total sample of patients, as well as separately for men and women, are given in Table 1.

**Table 1:** Descriptive statistics of the SPADI total score, pain and disability subscales, and Quick DASH and baseline characteristics of the study population

Patient characteristics	Total participants				Men		Women	
	Min (*n*)	Max (*n*)	(*n* = 102; 100 %)		(*n* = 42; 41.2 %)		(*n* = 60; 58.8 %)	
Age (years)	20 (3)	80 (1)	47.4	±14.5	48.3	±13.9t	46.7	±15.1
Weight (kg)	45 (1)	120 (1)	72.3	±15.3	83.8	±12.8	64.3	±11.2
SPADI pain subscale	12 (2)	95(2)	62.5	±16.2	59.8	±20.3	64.4	±12.5
SPADI disability subscale	0 (1)	96 (1)	43.3	±18.3	41.4	±20.9	44.8	±16.4
SPADI total	7 (1)	95 (1)	50.7	±16.4	48.4	±19.2	52.3	±14.1
Quick DASH	9 (1)	89 (1)	41.3	±14.6	40.4	±16.0	41.9	±13.6
Your health today EQ-5D	30 (1)	100 (1)	71.5	±17.3	73.6	±17.8	69.9	±16.9

Data are presented as mean ± SD and min–max values for total participants. Values in parenthesis indicate the frequencies of floor and ceiling values. There were no missing values

**Fig. 1:**
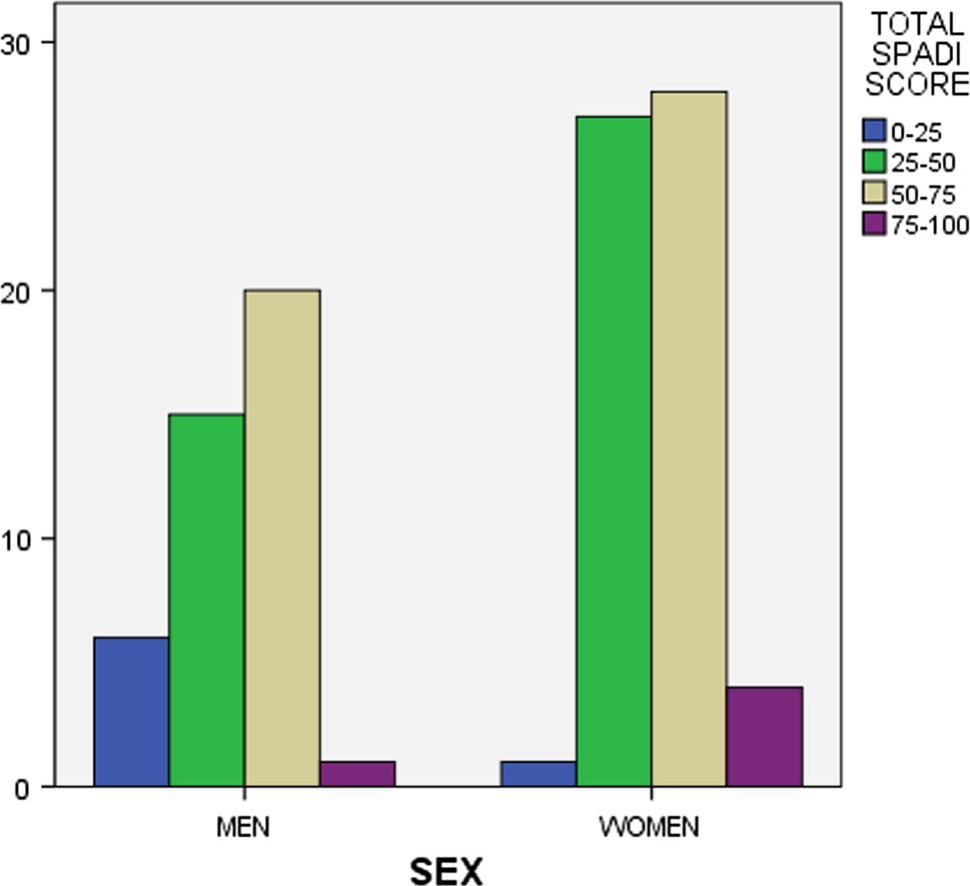
Distribution of gender according to SPADI score, separated into four subgroups (0–25, 25–50, 50–75, and 75–100)

**Fig. 2:**
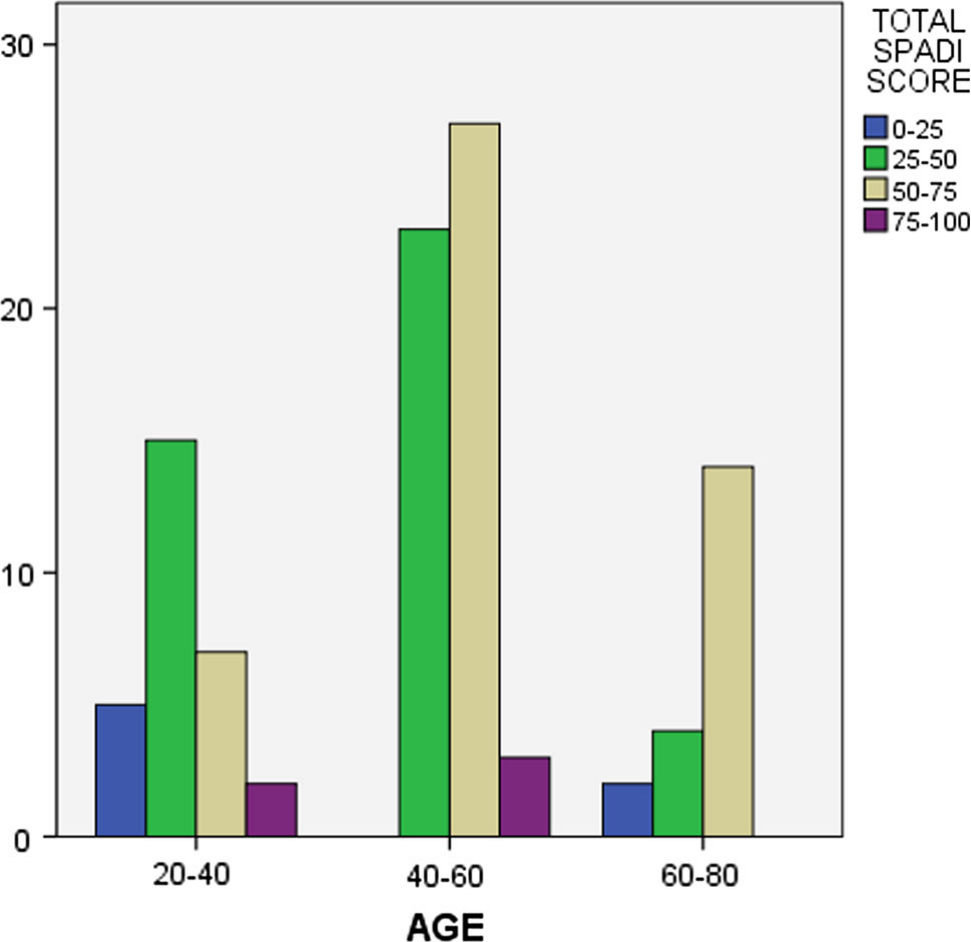
Distribution of age (divided into three subgroups: 20–40, 40–60, and >60 years old), according to SPADI score (divided into four subgroups: 0–25, 25–50, 50–75, and 75–100)

The mean score for the Pain subscale was 62.5 ± 16.2, for Disability 43.3 ± 18.3, and the mean total score for SPADI and Quick DASH was 50.7 ± 16.4 and 41.3 ± 14.6, respectively. No missing values were observed and no floor or ceiling effects were found. Women presented higher mean scores than men in SPADI (and its subscales) and in the Quick DASH, but these differences were not significant. Additionally, the majority of patients (>86 %) demonstrated SPADI total scores between 25 and 75, while almost the same proportion exhibited pain scores >50 %. On the other hand, more than 61 % of patients showed low disability scores, as shown in Table 2. Finally, 20 men (47.6 %) had total SPADI scores of 50–75 %, while the same scores were recorded from 46.7 % of women. An estimated 70 % of patients aged 60–80 years had total SPADI scores of 50–75 %, and more than half of patients younger than 40 years had lower scores. These results are presented in Figs. 1 and 2.

**Table 2:** Presentation of participants (*n*, %) after classification of Visual Analogue Scale scores (VAS, 0–100) into four categories (0–25, 25–50, 50–75, and 75–100)

Classification of VAS	Pain		Disability		SPADI total	
Range	*n*	%	*n*	%	*n*	%
[0–25)	4	3.9	14	13.7	7	6.9
[25–50)	10	9.8	49	48.0	42	41.2
[50–75)	72	70.6	37	36.3	48	47.1
[75–100)	16	15.7	2	2.0	5	4.9

### Reliability

The internal consistency was quite high for the SPADI scale (0.932). For the two subscales (Pain and Disability) Cronbach’s alpha coefficient was 0.905 and 0.899, respectively. Regarding the EQ-5D items, Cronbach’s alpha was 0.723. Item-to-total correlations ranged from 0.57 to 0.83, showing high correlations between the questions of the questionnaire. Reliability data are presented in Table 3.

**Table 3:** Cronbach’s alpha, item-to-total correlations, and intraclass correlation coefficients (ICCs) of the Greek version of the SPADI scale

	Number of items	Item-to-total correlations	Cronbach’s a	ICC	(95 % Confidence interval)
SPADI total	13	0.572–0.833	0.932	0.929	(0.907–0.948)
Pain	5	0.706–0.826	0.905	0.902	(0.868–0.929)
Disability	8	0.495–0.786	0.899	0.899	(0.866–0.926)
EQ-5D	5	0.3–0.665	0.723	0.723	(0.619–0.806)

Intraclass correlation coefficient (ICC) of SPADI subscales was found to be 0.902 (95 % confidence interval [CI] 0.868–0.929) for Pain and 0.899 (95 % CI 0.866–0.926) for Disability (i.e. the higher the number of items in the scale the higher the ICC), while a value of 0.929 (95 % CI 0.907–0.948) for the total SPADI score was found.

### Validity

The correlation between the scores of the SPADI subscales was quite high (*r* = 0.719, *p* < 0.01), as well as the correlations with the total SPADI score (*r* = 0.877 for Pain and *r* = 0.964 for Disability). We found a strong positive correlation between the Quick DASH and the SPADI total (*r* = 0.764, *p* <0.01), and the same applied to the SPADI subscales (*r* = 0.764 for Pain and *r* = 0.684 for Disability), as well as with the third variable of EQ-5D, “common activities” (*r* = 0.716, *p* <0.01).

The total SPADI scores were shown to have a significant relationship with each of the five EQ-5D variables: a moderate positive correlation with the variables “self-reliance” (*r* = 0.66), “common activities” (*r* = 0.58), and “pain/discomfort” *(r* = 0.49), and a weak correlation with the “mobility” variable (*r* = 0.20). No significant correlation was observed regarding the variable “anxiety/grief”. A moderate positive correlation was also observed between the Quick DASH “self-reliance” (*r* = 0.588) and “pain/discomfort” (*r* = 0.564). Correlations between the SPADI and its subscales with the Quick DASH and the five variables of EQ-5D are given in Table 4.

**Table 4:** Pearson correlattion coeficients for SPADI scores (total and subscales) with the Greek version of Quick DASH and the five variables of EQ-5D questionnaire

		Total pain score	Total disability score	Total SPADI score	Mobility	Self-reliance	Common activities	Pain/discomfort	Anxiety/grief	Your health today EQ-5D	Total Quick DASH
Total pain score	Pearson correlation	1	0.719**	0.877**	0.143	0.527**	0.575**	0.573**	0.193	−0.398**	0.764**
	Sig. (2-tailed)		0.000	0.000	0.150	0.000	0.000	0.000	0.052	0.000	0.000
	*N*	102	102	102	102	102	102	102	102	102	102
Total disability score	Pearson correlation	0.719**	1	0.964**	0.215*	0.674**	0.529**	0.401**	0.094	−0.394**	0.684**
	Sig. (2-tailed)	0.000		0.000	0.030	0.000	0.000	0.000	0.346	0.000	0.000
	*N*	102	102	102	102	102	102	102	102	102	102
Total SPADI score	Pearson correlation	0.877**	0.964**	1	0.203*	0.666**	0.584**	0.495**	0.139	−0.424**	0.764**
	Sig. (2-tailed)	0.000	0.000		0.040	0.000	0.000	0.000	0.164	0.000	0.000
	*N*	102	102	102	102	102	102	102	102	102	102
Mobility	Pearson correlation	0.143	0.215*	0.203*	1	0.214*	0.039	0.184	0.300**	−0.310**	0.101
	Sig. (2-tailed)	0.150	0.030	0.040		0.031	0.696	0.064	0.002	0.002	0.315
	*N*	102	102	102	102	102	102	102	102	102	102
Self-reliance	Pearson correlation	0.527**	0.674**	0.666**	0.214*	1	0.498**	0.315**	0.236*	−0.403**	0.588**
	Sig. (2−tailed)	0.000	0.000	0.000	0.031		0.000	0.001	0.017	0.000	0.000
	*N*	102	102	102	102	102	102	102	102	102	102
Common activities	Pearson correlation	0.575**	0.529**	0.584**	0.039	0.498**	1	0.608**	0.305**	−0.395**	0.716**
	Sig. (2-tailed)	0.000	0.000	0.000	0.696	0.000		0.000	0.002	0.000	0.000
	*N*	102	102	102	102	102	102	102	102	102	102
Pain/discomfort	Pearson correlation	0.573**	0.401**	0.495**	0.184	0.315**	0.608**	1	0.440**	−0.312**	0.564**
	Sig. (2-tailed)	0.000	0.000	0.000	0.064	0.001	0.000		0.000	0.001	0.000
	*N*	102	102	102	102	102	102	102	102	102	102
Anxiety/grief	Pearson correlation	0.193	0.094	0.139	0.300**	0.236*	0.305**	0.440**	1	−0.205*	0.345**
	Sig. (2-tailed)	0.052	0.346	0.164	0.002	0.017	0.002	0.000		0.039	0.000
	*N*	102	102	102	102	102	102	102	102	102	102
Your health today EQ-5D	Pearson correlation	−0.398**	−0.394**	−0.424**	−0.310**	−0.403**	−0.395**	−0.312**	−0.205*	1	−0.344**
	Sig. (2-tailed)	0.000	0.000	0.000	0.002	0.000	0.000	0.001	0.039		0.000
	*N*	102	102	102	102	102	102	102	102	102	102
Total quick DASH	Pearson correlation	0.764**	0.684**	0.764**	0.101	0.588**	0.716**	0.564**	0.345**	−0.344**	1
	Sig. (2-tailed)	0.000	0.000	0.000	0.315	0.000	0.000	0.000	0.000	0.000	
	*N*	102	102	102	102	102	102	102	102	102	102

*Sig*. significance

* Correlation is significant at the 0.05 level (2-tailed)

** Correlation is significant at the 0.01 level (2-tailed)

The results of the factor analysis showed that the choice of a factor explains 34.3 % of the total dispersion while the solution of two factors explains 63.6 %. The corresponding extraction communalities for the factor analysis ranged from 0.366 to 0.813, thus most of the variance of these variables was accounted for by this two-dimensional factor solution.

The individual loadings of questions (items) for these two factors are presented in Table 5. Eight items (five Pain and three Disability) weighted the first factor by a factor of >0.5, and five disability items weighted the second factor.

**Table 5:** Item-Total Statistics of the Greek SPADI score

	Scale mean if item deleted	Scale variance if item deleted	Corrected item-total correlation	Squared multiple correlation	Cronbach’s a if item deleted
Question 1	58.64	412.927	0.614	0.625	0.927
Question 2	59.71	404.051	0.618	0.708	0.926
Question 3	59.43	391.614	0.750	0.834	0.922
Question 4	60.75	386.167	0.757	0.726	0.921
Question 5	59.87	390.786	0.754	0.728	0.922
Question 6	62.07	374.540	0.725	0.777	0.923
Question 7	60.98	370.475	0.753	0.713	0.922
Question 8	62.19	363.262	0.740	0.737	0.923
Question 9	63.80	396.417	0.572	0.554	0.928
Question 10	63.40	392.678	0.589	0.481	0.927
Question 11	59.77	384.097	0.833	0.855	0.919
Question 12	59.60	403.589	0.575	0.619	0.927
Question 13	60.96	390.612	0.694	0.607	0.924

## Discussion

There are various scales used in clinical practice designed to elicit initial information about a disease, monitor the possible changes of symptoms, and evaluate the effectiveness of the therapeutic process [44]. The SPADI questionnaire has been used in multiple studies related to pain and disability of the upper limb [23–26, 28–31, 43, 45, 46], but until today it has not been translated and ratified in the Greek language. After translation and cultural adaptation of the SPADI questionnaire into Greek, its internal consistency (rated by Cronbach’s alpha) was calculated to be 0.929, a fact which is in accordance with current literature that considers values greater than 0.7 reliable [27, 36, 43, 47, 48]. The intraclass correlation coefficient (ICCs) was also high (>0.9), probably due to the short time between the first and second completion of the questionnaire. Bot et al. [49] reported that ICC values greater than 0.9 on a scale show that a tool is suitable for individual assessment of patients.

These results are consistent with those obtained by the original testing of the questionnaire in English, as demonstrated in several studies [29, 31, 32], as well as with the values obtained by testing the questionnaire in other languages [23, 24, 26, 28, 30, 45]. The internal consistency of the German SPADI was found to be 0.9 for the “pain”, 0.93 for the “disability/inability” and 0.95 to total German SPADI [23], versus 0.86, 0.93 and 0.95 of the original SPADI [20], respectively. MacDermid et al. [29] and Hill et al. [31] also reported a Cronbach’s alpha of >0.92 in each of the subscales for the English version of SPADI, a result that is in accordance with the study of Tveitå et al. [30], and with our results. Turkish researchers have also calculated a similar Cronbach’s alpha (0.83) for the subscales of pain and disability [27], while in the Arabic adaptation of the questionnaire the ICC value was calculated to be 0.96 and Cronbach’s alpha 0.911 also [25]. In the Indian version, the value of ICC was also 0.9 and Cronbach’s alpha 0.95 [26]. Finally, in the cultural adaptation process in the Brazilian population [24], it has been stated that in the test–retest reliability Cronbach’s alpha ranged between 0.90 and 0.94 and the internal consistency ranged between 0.87 and 0.89. These values were higher than those given by some researchers [20, 21, 49–54], and similar to others [23, 28].

In its original form, the SPADI lists 13 questions for “pain” and “disability” [20], but these two dimensions are not supported by all validity studies. The version in the Turkish language mentions three dimensions [27], whereas Tveitå et al. [30] in their study report that SPADI may be one-dimensional. Specifically, Tveitå et al. report that high ICC values and Cronbach’s alpha >0.9 and the analysis of the structure of the factors lead to the conclusion that the SPADI questions only assess “pain”, which is the main cause of functionality problems [30]. Pain as the main limitation for the implementation/execution of various activities, such as those recorded with the SPADI, is also described in other studies [55–61]. The factor analysis in our data revealed that eight items (five for pain and three for disability) are weighting the first factor, and five disability items are weighting the second factor. Therefore, we are leaning towards the proposal of the two-dimensional SPADI scale (for “pain” and “disability”), as well as the original development of the questionnaire [20], despite the fact that the last three questions (“when placing an object on a high shelf”, “when lifting an object of weight 4.5 kg”, “when you want to take something from your back pocket”) seem to be allocated to the first factor, “pain”. This suggests that the individuals included in our sample had a better or greater perception of pain, despite their functional limitation. Other studies also converged to the same conclusion, but with variations in the questions which are included in the two dimensions [24, 29, 31].

Another interesting observation from this study was that women exhibited higher total pain scores compared to men, and higher levels in disability scores. Generally, in musculoskeletal problems, women tend to report pain more often, with longer duration and greater severity, in comparison with men [62, 63]. The perception of disability in women as a result of rupture of the rotator cuff appears to be influenced by social factors [64]. The role of women, both in the family (providing care) and at work (fulfillment of similar work to their male colleagues), pushes them further towards a declaration of incapacity [64]. Gialanella et al. [65], in a study regarding the ability to work at home in housewives with a total thickness rotator cuff tear, found that 84 % required help to perform some activities, such as vacuuming. In addition to women, patients of younger age also presented with more intense pain in comparison with the overall sample [64, 66, 67]. This can be explained by the fact that natural function attenuates with increasing age [67], and therefore less workload is applied. Often, this is not compatible with old age per se, but with the presence of a rotator cuff tear [67]. In our study, no one in the age group of 60–80 years reported disability scores over 75 %, maybe because of lower functional status generally in older populations.

The necessity of measurement of health-care needs and assessment of health status by using different social, economic, and psychological indicators imposed the use of the EQ-5D and the Quick DASH concomitantly with the SPADI [36]. The SPADI questionnaire appeared to correlate directly with the Quick DASH scale and with three of the five components of the EQ-5D: specifically, a moderate positive correlation with self-care, usual activities, and pain/discomfort was observed. This is consistent with studies related to chronic shoulder pain and several disorders of the rotator cuff [1]. No association was observed in our results with the mobility factor of the EQ-5D, obviously because it referred to the ability of patients to walk. Furthermore, no statistical relationship was revealed between the total SPADI score and the factor “anxiety/sadness” [47].

A main limitation of our results is that the sample size would have been higher if all of the patients who had been given the questionnaire answered all of its questions. Specifically, 102 of the 134 questionnaires were considered valid. Another limitation is the absence of a correlation between SPADI and other questionnaires, since several tools have the ability to elicit different aspects of pain and functionality regarding the same pathology [29].

To conclude, a satisfactory test–retest reliability, internal consistency, and construct and structural validity were displayed by this study of the Greek version of the SPADI questionnaire. Therefore, it represents a reliable and valid tool that can record the pain and incapacity caused by shoulder pain in the Greek population. This translation and cultural adaptation of the SPADI questionnaire, in addition to its validation in patients with rotator cuff tears, will significantly help Greek scholars and researchers to obtain data regarding disorders of the shoulder, and to design new studies for improving treatment and the quality of patients’ lives. However, further research is required in this area in order to validate the SPADI questionnaire in other shoulder diseases and patient populations.
